# Stachydrine Ameliorates Cardiac Fibrosis Through Inhibition of Angiotensin II/Transformation Growth Factor β1 Fibrogenic Axis

**DOI:** 10.3389/fphar.2019.00538

**Published:** 2019-05-22

**Authors:** Xiao Liu, Xiaoli Shan, Huihua Chen, Zan Li, Pei Zhao, Chen Zhang, Wei Guo, Ming Xu, Rong Lu

**Affiliations:** ^1^Department of Integrated Chinese and Western Medicine, School of Basic Medical Sciences, Shanghai University of Traditional Chinese Medicine, Shanghai, China; ^2^Experimental Center, School of Basic Medical Sciences, Shanghai University of Traditional Chinese Medicine, Shanghai, China; ^3^Department of Physiology, School of Basic Medical Sciences, Shanghai University of Traditional Chinese Medicine, Shanghai, China; ^4^Department of Pathology, School of Basic Medical Sciences, Shanghai University of Traditional Chinese Medicine, Shanghai, China

**Keywords:** cardiac fibrosis, cardiac fibroblasts, myofibroblasts, angiotensin II, transformation growth factor-β1, Smads

## Abstract

Cardiovascular diseases, the leading cause of death worldwide, are tightly associated with the pathological myocardial fibrosis. Stachydrine (Sta), a major active compound in Chinese motherwort *Leonurus heterophyllus*, was reported to effectively attenuate cardiac fibrosis, but the cellular and molecular mechanism remains unclear. In this study, the anti-fibrotic effect of Sta and mechanism underlying were explored in a mouse model of pressure overload and AngII stimulated cardiac fibroblasts (CFs). Mice were randomly divided into sham, transverse aorta constriction with saline (TAC+Sal), TAC with telmisartan (TAC+Tel), and TAC with Sta (TAC+Sta) groups. Cardiac morphological and functional changes were evaluated by echocardiography and histological methods, and the molecular alterations were detected by western blotting. Primary cultured neonatal mouse CFs were treated with or without angiotensin II (AngII, 10^−7^ M), transformation growth factor β1 (TGFβ1, 10 ng/mL), and different dosage of Sta (10^−6^–10^−4^ M) for up to 96 h, and cell proliferation, cytotoxicity, morphology and related signals were also detected. The *in vivo* results revealed that TAC prominently induced cardiac dysfunction, left ventricular dilation, myocardial hypertrophy, and elevated myocardial collagen deposition, accompanied with increased fibrotic markers including α-smooth muscle actin (α-SMA) and periostin. However, Sta treatment partially reversed cardiac morphological and functional deteriorations, and significantly blunted cardiac fibrosis as well as Tel. Increments of myocardial angiotensinogen (AGT), angiotensin converting enzyme (ACE), AngII type 1 receptor (AT1R), and TGFβ1 transcripts, together with increased protein levels of ACE and AngII, after TAC were dramatically down-regulated by Sta treatment. Coincidently, *in vitro* experiments demonstrated that AngII stimulation in CFs led to up-regulation of AT1R and TGFβ1, and therefore promoted CFs *trans*-differentiating into hyper-activated myocardial fibroblasts (MFs) as evidenced by increased cell proliferation, collagen and fibrotic makers. On the contrary, Sta potently down-regulated but not directly inhibited AT1R, suppressed TGFβ1 production, and the pro-fibrotic effect of AngII in CFs. Moreover, activation of TGFβ1/Smads signal in the fibrotic process were observed both TAC model and *in* AngII stimulated CFs, which were also notably blunted by Sta. However, Sta failed to abolish the activation of CFs triggered by TGFβ1. Taken together, it was demonstrated in this study that Sta suppressed ACE/AngII/AT1R-TGFβ1 profibrotic axis, especially on the *de novo* production of AngII via down-regulating AGT/ACE and AT1R, and therefore inactivated CFs and blunted MFs transition, which ultimately prevented cardiac fibrosis.

## Introduction

Cardiovascular diseases (CVDs) is the leading cause of death in the world, which has become a major burden for the global health nowadays. According to “China’s cardiovascular health index (2017),” it has been proposed that over 290 million people are suffering from CVDs, accounting for 40% of the total number of deaths in china. Cardiac fibrosis, characterized by net accumulation of extracellular matrix (ECM) proteins, is a common pathophysiological change of most myocardial diseases. It is tightly associated with cardiac dysfunction, arrhythmia, and adverse clinical outcome, and interventions targeting fibrotic activation via modulating the composition of the interstitial ECM has been proved to have profoundly beneficial effect (Frangogiannis, 2018).

Disturbance of ECM homeostasis increases ventricular wall stiffness, uncouples myocardial excitation-contraction coupling via disruption of the link between the matrix and the sarcomere, and induces dysrhythmias by perturbing conduction of the electrical impulse, which ultimately results in functional abnormalities in the diseased heart (Spinale, 2007). The key cellular event during the myocardial fibrotic response is the activation and *trans*-differentiation of fibroblasts into myofibroblasts (MFs) that persistently exert the fibrogenic properties including hyperactive synthesis and secretion of collagens, contractility, and positive feedback activation of fibroblasts and MFs by their paracrine or autocrine actions (Hinz, 2010; Weber et al., 2013; Travers et al., 2016; Weber and Diez, 2016). Besides the mechanical stretch and the injured cells in the diseased heart, a wide range of bioactive mediators, including local renin–angiotensin–aldosterone system (RAAS), cytokines such as transforming growth factor β1 (TGFβ1) and interleukin-10 (IL-10), and matricellular proteins, play an important role in modulating the fibroblast behavior (Leask, 2010; Segura et al., 2014; Wu et al., 2017).

Among these bioactive stimuli, angiotensin II (AngII) and TGFβ1 are the mostly dominant molecules in cardiac fibrosis. It has been well established by Weber et al. (2013) and Weber and Diez (2016) that local AngII and its subsequent autocrine action via binding to the AngII type 1 receptor (AT1R) maintain the MFs activity such as collagen synthesis and TGFβ1 generation. Additionally, TGFβ1 enhances matrix proteins deposition and inhibits ECM degradation via Smad-dependent or Smad-independent pathways (Horiguchi et al., 2012). Moreover, extensive clinical and experimental evidence supports the protective effect of angiotensin converting enzyme inhibitor (ACEI) and AT1R blocker against cardiac fibrosis (Brilla et al., 1991; Varo et al., 2000; Diez et al., 2002; Ciulla et al., 2004).

The Chinese motherwort *Leonurus heterophyllus* Sweet has been widely applied in traditional medicine to promote blood circulation and dispel blood stasis, and mostly in the treatment of gynecological and obstetrical disorders. Recent studies has demonstrated that *Leonurus heterophyllus* Sweet exhibits multiple cardioprotective effects by its antioxidant, anti-apoptotic, angiogenic, and anti-platelet aggregation activities (Liu et al., 2007). Coincidently, Stachydrine (Sta), a major active betaine compound in it, was also reported to elicit such effects (Yin et al., 2010; Guo et al., 2012; Servillo et al., 2013; Zhang et al., 2014; Cao et al., 2017; Xie et al., 2018). In addition, our recent study discovered that Sta treatment attenuated myocardial fibrosis via TGFβ1/Smad pathway in a rat model of heart failure induced by pressure overload (Chen et al., 2017). Meanwhile, Zhao et al. (2017) also reported the anti-fibrotic effect of Sta through NF-κB and JAK/STAT signaling pathways in a rat model of isoproterenol induced cardiac remodeling. However, these studies only revealed a preliminary mechanism underlying the anti-fibrotic effect of Sta, and the intrinsic cellular changes and molecular pathways involved still remains unclear. Hence, a mouse model of pressure overload and cardiac fibroblasts (CFs) stimulated by AngII were applied in this study to further explore the effect and mechanism of Sta on cardiac fibrosis.

## Materials and Methods

### Animals and Drugs

Male adult C57BL/6 mice (8 weeks, mean body mass 22.3 ± 1.2 g) were purchased from Shanghai Laboratory Animal Center (Shanghai, China). All procedures in this study were approved by the Animal Care and Use Committee of Shanghai University of Traditional Chinese Medicine, which is in accordance with the National Institutes of Health guide for the care and use of laboratory animals (NIH Publications No. 8023, revised 1978). The animals were housed in cages under standard conditions at 25°C with a 12/12 h light-dark cycle and allowed free access to water and standard diet. Sta [2-carboxy-1,1-dimethyl-, chloride (1:1), (2S)-pyrrolidinium, C7H14NO2.Cl, ≥98% purity] was purchased from Tauto Biotech Co., Ltd. (Shanghai, China), which was extracted from *Leonurus sibiricus*. Telmisartan, AngII (Sigma-Aldrich, MO, United States) and TGFβ1 (Cell Signaling Technology, Shanghai, China) were dissolved in saline for *in vivo* experiment and distilled water for *in vitro* experiment.

### Transverse Aortic Constriction

Transverse aorta constriction (TAC) surgery was used to generate cardiac hypertrophy and heart failure model. Briefly, general anesthesia was induced with 3% of isoflurane and an oxygen flow of 1 L/min by a calibrated vaporizer (Surgivet Inc., WI, United States), and maintained by mechanical ventilation (150 bpm, 250 μL tidal volume, 2% isoflurane and an oxygen flow of 1 L/min) (Harvard Inspira-ASV, Harvard Apparatus, MA, United States). Mice were placed in a supine position on a warm heating pad (World Precision Instruments Inc., FL, United States). After thoracotomy, the aortic arch was carefully dissected free of surrounding tissues, and a bended and blunted stylet from a 27G intravenous catheter was tightly tied to the aorta between the brachiocephalic trunk and the left common carotid artery using 7-0 silk, and then removed to create partial aortic constriction. The sternum and the skin incision were closed with 5-0 sutures. The sham group underwent all operation procedures except the ligation of aorta. Postoperative animals were kept in separated cages and allowed free access to water and standard diet. Analgesia with buprenorphine 0.05 mg/kg subcutaneously was provided every 12 h for 48 h.

### *In vivo* Medication Treatment

After 3 days of operation, animals were randomly divided in to sham, TAC plus saline (TAC+Sal), TAC plus telmisartan (TAC+Tel), and TAC plus Sta (TAC+Sta) groups (*n* = 6∼8 in each group), and the whole duration for this study lasted for 4 weeks. The sham and TAC groups were treated with saline, while the TAC+Tel and TAC+Sta groups were treated with Tel (7.2 mg/kg, a selective AT1R antagonist as positive control) and Sta (12 mg/kg) once a day by oral gavage, respectively (Chen et al., 2017; Miao et al., 2017; Zhao et al., 2017; Zhang et al., 2018).

### Echocardiography

Echocardiographic assessment was performed by a high resolution ultrasound imaging system (VINNO 6, Vinno Corporation, Suzhou, China) with a 23 MHz probe. The animals were anesthetized with 1.5% isoflurane in 95% oxygen and 5% carbon dioxide, and the hair was removed with depilatory cream 1 day before the examination. M-mode recordings were obtained from the parasternal short axis views. The internal dimensions of left ventricular (LV) cavity, left ventricular posterior wall (LVPW) and interventricular septal wall (IVS) thickness, LV fractional shortening (FS), and LV ejection fraction (EF), heart rate (HR), cardiac output (CO), and stroke volume (SV) were measured and recorded.

### Histological and Immunofluorescence Procedure

Tissue samples were fixed in 4% paraformaldehyde overnight at 4°C, rinsed, transferred to phosphate buffer saline (PBS), and embedded by paraffin. Masson’s trichrome staining was performed, and collagen deposition was quantitatively analyzed by collagen volume fraction via Image Pro Plus (Media Cybernetics Inc., MD, United States) after the sections were examined by light microscopy and photographed.

Alexa Fluor^TM^ 594 conjugated wheat germ agglutinin (WGA, Thermo Fisher Scientific, Shanghai, China) labeling was applied to detect the cross sectional areas (CSA) of the cardiomyocytes according to the manufacture’s instruction. Mean CSA of the cardiomyocytes was quantified by the ImageJ.

For cellular immunofluorescence, cells were plated on glass cover slips, fixed with 70% ethanol for 20 min, and incubated with 2% bovine serum albumin (BSA) for 20 min at room temperature to reduce non-specific binding. Then followed by overnight incubation at 4°C with primary antibodies against α smooth muscle actin (α-SMA) (Abcam, Shanghai, China), anti-phosphorylated Smad2/3, anti-smad4 (Cell Signaling Technology, shanghai, China) in PBS containing 2% BSA. Secondary antibodies coupled with Alexa Fluor 488 (Invitrogen, CA, United States) was used, and 4′-6-diamidino-2-phenylindole (DAPI, Beyotime, Beijing, China) was chosen for cell nuclei staining. After washing with PBS, the slides were mounted in 50% glycerol and 50% PBS. Cover slips were mounted onto slides by fluorescence mounting medium (Dako, Beijing, China) and observed under an inverted fluorescence microscope from Olympus.

### Isolation and Culture of Neonatal CFs

Primary CFs were isolated from the hearts of 1- to 3-day-old neonatal C57BL/6 mice with 0.25% trypsin (Wang et al., 2018). Fibroblasts were maintained in high-glucose Dulbecco’s modified Eagle’s medium (DMEM, Thermo Fisher, Shanghai, China) supplemented with 10% fetal bovine serum (FBS, Thermo Fisher, Shanghai, China), 100 U/mL penicillin, and 100 mg/mL streptomycin (Life Technologies, Carlsbad, CA, United States). The fibroblasts were used for experiments after the second passage. After starvation in DMEM with 1% FBS overnight, CFs were treated with or without AngII (10^−7^ M, Sigma-Aldrich, MO, United States) or TGFβ1 (10 ng/mL), together with or without Sta (10^−6^–10^−4^ M), for different time periods.

### Real-Time Cell Proliferation Index and LDH Releasing Assay

The xCELLigence system (ACEA, biosciences, Inc., Hangzhou, China) was used according to the manufacturer’s instructions. An impedance-based real time cell analyzer (RTCA), an RTCA single plate (E-plate 96), an RTCA computer, and a tissue-culture incubator constitute the xCELLigence system (Mendi et al., 2017). Adherent cells are determined and measured by the electronic impedance of the sensor electrodes, which was indicated as cell index. Briefly, second passage of CFs were inoculated at a concentration of 1 × 10^5^/mL with complete medium for 3 h, followed by starvation in DMEM with 1% FBS for 2 h, and then treated with or without AngII (10^−7^ M), TGFβ1 (10 ng/mL), together with or without Sta (10^−6^–10^−4^ M). Cell index was monitored and recorded every 30 min for up to 96 h.

Lactate dehydrogenase (LDH) level released from cells was detected by LDH-Cytotoxic Assay Kits (Beyotime Biotechnology, Nanjing, China) to assess cytotoxicity after drug treatment according to the manufacturer’s instructions. The medium without cells was served as a blank control. The LDH content in the medium was calculated based on the absorbance which was normalized with the blank control. The average LDH level was normalized by OD value representing cell numbers.

### Western Blotting

Protein lysates from heart samples were loaded on and separated by a 10–15% SDS-PAGE, and then transferred to PVDF membranes (Millipore, MA, United States). The membranes were probed with primary antibodies overnight at 4°C after blocking with 5% milk in Tris-buffered saline with 0.1% Tween (TBST). The primary antibodies were anti-TGFβ1, anti-TGFβ receptor I (TGFβRI), anti TGFβ receptor II (TGFβRII), anti-phosphorylated Smad2/3, anti-Smad2/3, and anti-Smad4 (Cell Signaling Technology, shanghai, China), anti-collagen I and III (Col I and III, Proteintech, Wuhan, China), anti-α-SMA, anti-periostin, anti-ACE (Abcam, Shanghai, China) and anti-glyceraldehyde-3-phosphate dehydrogenase (GAPDH) (Multi Sciences, Hangzhou, China). Membranes were washed by TBST for three times and then incubated with appropriate secondary antibodies for 1 h at room temperature. After another three times wash by TBST, the signal was detected on FluorChemE (Protein Simple, CA, United States). The densities of bands were quantified by an ImageJ Analysis System and expressed as ratios to GAPDH.

### RNA Analysis

Total RNA was extracted from heart tissue or CFs with TRIzol reagent (Invitrogen, CA, United States), and reverse transcribed with SuperScript reverse transcriptase kit (Invitrogen, CA, United States). Gene expression was analyzed by quantitative real-time polymerase chain reaction (PCR) using SYBR^^®^^ dye (LightCycler^^®^^ 96 Real-Time PCR System, Roche, Switzerland). Transcript quantity was determined by relative value to the reference 18S rRNA and normalized to the mean value of samples. The primers encoding *Mus musculus* angiotensinogen (AGT), ACE, AT1R, TGFβ1, and 18S were as follows:

**Table:** 

AGT	F	GACAGCACCCTACTTTTCAACAC
	R	TCTATCCAAGTCAGGAGGTCGTT
ACE	F	CAGACAACAACTCACCAAGCAAC
	R	TCTGCGTACTCGTTCAACAACAC
TGFβ1	F	AACAACGCCATCTATGAGAAAAC
	R	GTAACGCCAGGAATTGTTGCTAT
18S	F	GTAACCCGTTGAACCCCATT
	R	CCATCCAATCGGTAGTAGCG

### AngII Enzyme Linked Immunosorbent Assay

The levels of AngII in myocardial and cell culture media were detected by ELISA assay using commercial ELISA kits (Nanjing Jiancheng Bioengineering Institute, Nanjing, China) according the manufacturer’s instructions.

### *In vitro* Assessment of AngII Receptor Binding Assay

The receptor binding assay was conducted on HEK293 cell membranes. To obtain cell membranes, HEK293 cells were cultured to 90% confluence in DMEM with 15% FBS, collected and homogenized in an ice-cold buffer containing 0.25 mol/L sucrose, 5 mmol/L Tris/HCl, 5 mmol/L EDTA, pH 7.4, and a protease inhibitor cocktail (Complete, Boehringer Mannheim, Germany). The suspension was centrifuged at 50,000 × *g* for 30 min at 4°C, and the pellet was resuspended in a binding buffer containing 50 mmol/L Tris/HCl and 5 mmol/L EDTA at pH 7.4. After a second time centrifugation as described above, the membrane pellet was finally resuspended in the binding buffer at 1 mg of protein/mL, aliquoted, quick frozen in liquid N_2._ Aliquots containing 15 mg of protein were incubated at 25°C for 1 h in incubation buffer containing (final concentrations): NaCl (120 mM), MgCl_2_ (5 mM), 0.006% BSA, and Tris (50 mM), adjusted to pH 7.5. Incubation was initiated by the addition of 0.1 nM AngII (5-l-isoleucine) tyrosyl-^125^I-monoiodinated (Sar1-Ile8-AngII [^125^I], PerkinElmer, Waltham, MA, United States). Total incubation volume was 250 μL. Non-specific binding was measured by incubation in the presence of 5 nM AngII. Sta and losartan were studied in the range of concentrations 3.91 × 10^−9^∼1.024 × 10^−3^ M and 1.52 × 10^−10^∼3 × 10^−6^ M, respectively. Binding was terminated by rapid filtration using a Millipore multi-screen device. Filters were washed three times with 250 μL of the corresponding buffer. Dry filters were placed into vials containing 3 mL of scintillation fluid, and the radioactivity was counted in a scintillation counter. The IC_50_ value (concentration for 50% displacement of the specifically bound Sar1-Ile8-AngII [^125^I]) was estimated from the linear portion of the displacement curve. Assays were performed in triplicate.

### Statistical Analysis

All values were analyzed with SPSS18.0 and results were presented as means ± SEM. The data were analyzed with one-way analysis of variance (one-way ANOVA) followed by Tukey’s *post hoc* analysis, and *P*-value less than 0.05 was considered statistically significant.

## Results

### Sta Treatment Alleviated Pressure Overload Induced Cardiac Morphological Remodeling and Pulmonary Congestion

**FIGURE 1:**
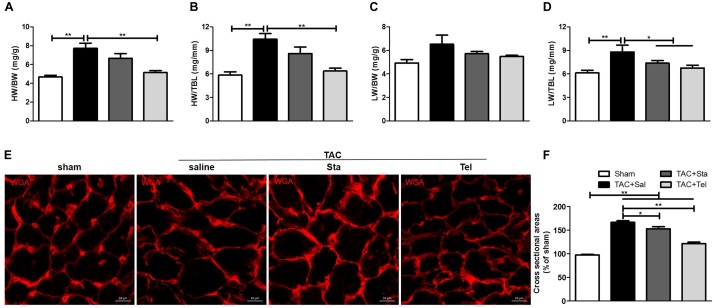
Sta treatment alleviated pressure overload induced cardiac morphological remodeling and pulmonary congestion. **(A,B)** Quantification of heart weight-to-body weight ratio and heart weight-to-tibial length ratio. **(C,D)** Quantification of lung weight-to-body weight ratio and lung weight-to-tibial length ratio. **(E)** Representative immunofluorescence images of cross sectional area of cardiomyocyte by WGA (magnification ×100), scale bar: 10 μm. **(F)** Mean cross sectional area were quantified by the image-J cell area measurement software, and at least 30 cardiomyocytes per animal in each group were measured. Animal heart samples were collected at 4 weeks after surgery. The data were expressed as the mean ± SEM. ^∗∗^*P* < 0.01; ^∗^*P* < 0.05; *n* = 6–8 in each group; TAC, transverse aorta constriction; Sal, saline; Sta, stachydrine; Tel, telmisartan; HW, heart weight; BW, body weight; TBL, tibial length; WGA, wheat germ agglutinin.

Transverse aorta constriction is a common procedure used to induce left ventricular remodeling by means of an increased cardiac afterload. In this study, the cardiac morphological remodeling was first assessed after 4 weeks of the surgery. As shown in Figure 1A,B, the TAC+Sal group exhibited significantly increased heart weight to body weight and heart weight to tibial length ratios (*P* < 0.01 vs. the sham group). Accordingly, pulmonary congestion was detected after TAC, evidenced by augment of lung weight to tibial length ratios (*P* < 0.01 vs. the sham group, Figure 1D). Administration of Tel markedly reduced heart weight to body weight and heart weight to tibial length ratios (*P* < 0.01 vs. the TAC+Sal group, Figure 1A,B), but treatment of Sta only showed a trend of that reduction (*P* > 0.05 vs. the TAC+Sal group, Figure 1A,B). Moreover, pulmonary congestion induced by TAC was notably alleviated by Tel or Sta treatment (*P* < 0.05 vs. the TAC group, Figure 1D), however the lung weight to body weight data only revealed a trend of such amelioration (*P* > 0.05 among all groups, Figure 1C). The CSA of the cardiomyocyte further verified that hypertrophied cardiomyocyte after TAC were partially reversed by Sta or Tel treatment (*P* < 0.05 or *P* < 0.01 vs. the TAC+Sal group, Figure 1E,F). These data provided that Sta potently benefited pressure overload induced cardiac remodeling via alleviation of myocardial hypertrophy and subsequent pulmonary congestion.

### Sta Attenuated Pressure Overload Induced Cardiac Functional Remodeling

**FIGURE 2:**
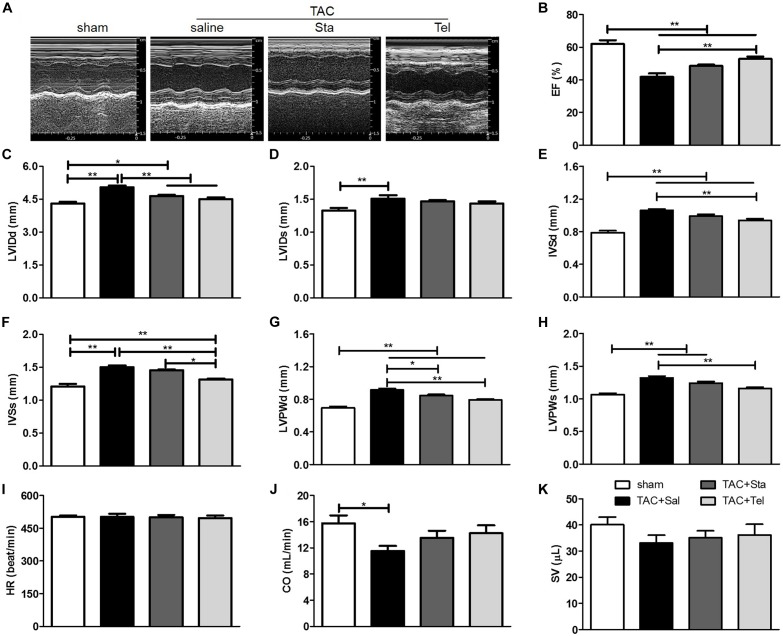
Sta attenuated pressure overload induced cardiac functional remodeling. **(A)** Representative M-mode tracings of echocardiography after 4 weeks of surgery. **(B–K)** Quantification echocardiographic parameters: **(B)** Left ventricular ejection fractions (EF); **(C)** left-ventricular internal dimension at diastole (LVIDd); **(D)** left-ventricular internal dimension at systole (LVIDds); **(E)** interventricular septal wall thickness at diastole (IVSd); **(F)** interventricular septal wall thickness at systole; **(G)** left ventricular posterior wall thickness at diastole (LVPWd); **(H)** left ventricular posterior wall thickness at systole (LVPWs); **(I)** heart rate (HR); **(J)** cardiac output (CO); **(K)** stroke volume (SV). The data were expressed as the mean ± SEM. ^∗∗^*P* < 0.01; ^∗^*P* < 0.05; *n* = 6–8 in each group; TAC, transverse aortic constriction; Sal, saline; Sta, stachydrine; Tel, telmisartan.

To further evaluate the *in vivo* effect of Sta on TAC induced cardiac functional alteration, echocardiography was applied at the same heart rate of approximately 500 beat per minute (*P* > 0.05 among all the groups, Figure 2I). As shown in Figure 2A–D,J, pressure overload elicited significant enlarged LV chamber size, reduced LVEF and CO in comparison with those in the sham group (*P* < 0.05 vs. the sham group). Improved LVEF was only detected in the TAC+Tel group (*P* < 0.01 vs. the TAC+Sal group, Figure 2A,B) but not in the TAC+Sta group (*P* > 0.05 vs. the TAC+Sal group, Figure 2A,B), and both Sta and Tel treatment showed a trend to improved CO or SV (*P* > 0.05 vs. the TAC+Sal, Figure 2A,J,K). Notably, less dilated left ventricle chamber size was discovered in both the TAC+Tel and TAC+Sta groups (*P* < 0.01 vs. the TAC+Sal group, Figure 2A,C). In addition, the parameters of wall thickness showed that Sta partially alleviated LV wall hypertrophy caused by TAC (*P* < 0.05 vs. the TAC+Sal group, Figure 2A,G), which is not as strong as the effect of Tel (*P* < 0.01 vs. the TAC+Sal group, Figure 2A,E–H). These data implied that Sta, as well as Tel, protected heart from pressure overload induced functional deterioration.

### Sta Suppressed Myocardial Fibrosis Induced by Pressure Overload

**FIGURE 3:**
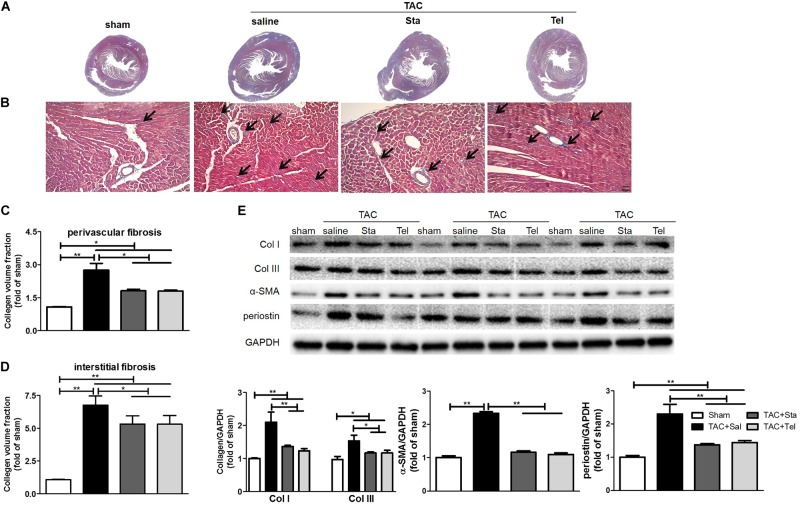
Sta suppressed myocardial fibrosis induced by pressure overload. **(A)** Representative slides of cardiac cross sections by Masson’s trichrome. **(B)** Representative histology of cardiac interstitial fibrosis by Masson’s trichrome staining (magnification ×100). The cardiomyocytes were stained for red, and collagenous fibers were blue. Scale bar: 20 μm. **(C,D)** Quantification of cardiac perivascular and interstitial fibrosis. **(E)** Representative western blots (upper panel) and quantification (lower panel) of myocardial Col I and III, α-SMA, periostin, and GAPDH. Collagen deposition was quantitatively analyzed via collagen volume fraction, which equals collagen area/total area × 100%. At least five to six slides per animal in each group were measured by Image-Pro Plus. The data were expressed as the mean ± SEM. ^∗∗^*P* < 0.01; ^∗^*P* < 0.05; *n* = 3–4 in each group; TAC, transverse aortic constriction; Sal, saline; Sta, stachydrine; Tel, telmisartan; Col I and III, collagen I and III; α-SMA, α-smooth muscle actin; GAPDH, glyceraldehyde-3-phosphate dehydrogenase.

The cardiac fibrosis contributes greatly to the cardiac remodeling process, and ultimately leads to cardiac dysfunction. In consideration of this and our previous work (Chen et al., 2017), cardiac collagen deposition was then measured by Masson’s trichrome staining. As illustrated in Figure 3A, obvious perivascular and interstitial fibrosis in the cross section of the heart samples from the TAC+Sal group was observed (*P* < 0.01 vs. the sham group, Figure 3A–D). The treatment of Sta, as well as Tel, notably suppressed TAC induced cardiac fibrosis to almost the same extent (*P* < 0.05 vs. the TAC+Sal group, Figure 3A–D). Additionally, the fibrotic markers, including collagen I and III, α-SMA and periostin (Snider et al., 2009; Kanisicak et al., 2016), significantly elevated after 4 weeks of TAC (*P* < 0.01 or *P* < 0.05 vs. the sham group, Figure 3E), which were markedly suppressed by Sta or Tel (*P* < 0.01 or *P* < 0.05 vs. the TAC+Sal group, Figure 3E). These findings implied that Sta, as well as Tel, might protected heart from pressure overload induced cardiac remodeling by the anti-fibrotic effect.

### Sta Alleviated Pressure Overload Induced Cardiac Fibrosis by Down-Regulation of AngII and TGFβ1/Smads Signaling Cascade

**FIGURE 4:**
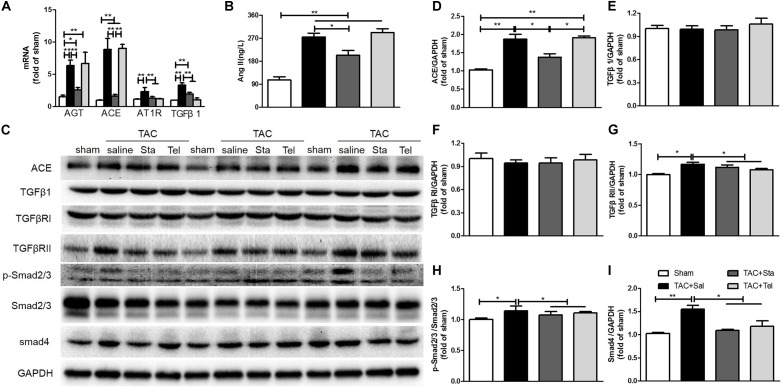
Sta alleviated pressure overload induced cardiac fibrosis by down-regulation of AngII and TGFβ1/Smads signaling cascade. **(A)** Quantitative real-time PCR of myocardial ATG, ACE, AT1R, and TGFβ1. **(B)** Quantification of myocardial AngII by ELISA. **(C)** Representative western blots of myocardial ACE, TGFβ1, TGFβRI, TGFβRII, Smad4, GAPDH, phosphorylated and total Smad2/3. **(D–I)** Quantification of myocardial ACE, TGFβ1, TGFβRI, TGFβRII, Smad4, phosphorylated and total Smad2/3. The data were expressed as the mean ± SEM. ^∗∗^*P* < 0.01; ^∗^*P* < 0.05; *n* = 3–4 in each group; TAC, transverse aorta constriction; sal, saline; Tel, telmisartan; Sta, stachydrine; AGT, angiotensinogen; ACE, angiotensin converting enzyme; AT1R, angiotensin type 1 receptor; AngII, angiotensin II; ELISA, enzyme linked immunosorbent assay; TGFβ1, transforming growth factor β1; TGFβRI, TGFβ receptor I; TgfRII, TGFβ receptor II; GAPDH, glyceraldehyde-3-phosphate dehydrogenase.

AngII, TGFβ1, endothelin-1 (ET-1), other chemicals, and mechanical stimuli modulate cardiac fibrosis, which thereafter activate the signaling axes including canonical TGFβ/Smads, non-canonical TGFβ/MAPKs, actin cytoskeleton, and calcium dependent pathways, and thus result in the activation and differentiation of fibroblasts (Moustakas and Heldin, 2009; Sciarretta et al., 2009; Teekakirikul et al., 2010; Stempien-Otero et al., 2016). In addition, the fibrogenic axis consisting ACE/AngII/AT1R-TGFβ1 was reported to be a main molecular mechanism underlying the transdifferentiation of fibroblasts (Weber et al., 2013). Here, we focused on the *de novo* production of AngII and TGFβ1 signaling pathway to illustrate the *in vivo* mechanism underlying the anti-fibrotic effect of Sta. Pressure overload forcefully elevated the transcriptional levels of myocardial AGT, ACE, AT1R, and TGFβ1 (*P* < 0.01 vs. the sham group, Figure 4A), while Sta significantly reduced all these transcripts (*P* < 0.01 vs. the TAC+Sal group, Figure 4A). Coincidently, both myocardial AngII and ACE protein levels were significantly elevated after TAC (*P* < 0.01 vs. the sham group, Figure 4B–D), which were notably inhibited by Sta (*P* < 0.05 vs. the TAC+Sal group, Figure 4B–D). Although Tel elicited no effect on the transcriptional and protein levels of elevated AGT, ACE and AngII induced by TAC (*P* > 0.05 vs. the TAC group, Figure 4A–D), it still notably reduced the AT1R and TGFβ1 transcripts (*P* < 0.01 vs. the TAC group, Figure 4A).

Even though the protein levels of TGFβ1 and TGFβ1RI in all the groups kept unchanged (*P* > 0.05, Figure 4C,E,F). Up-regulation of TGFβRII, Smad4 and obviously phosphorylated Smad2/3 (*P* < 0.05 vs. the sham group, Figure 4C,G–I) after TAC were significantly down-regulated after Sta or Tel treatment (*P* < 0.05, Figure 4C,G–I). These data implied that pressure overload elevated *de novo* production of local AngII and subsequently activated TGFβ1 signaling pathways, while the application of Sta not only suppressed the production of AngII but TGFβ1/Smads signaling pathways as well.

### Sta Suppressed AngII Activated Cardiac Fibroblast-Myofibroblast Transition via Inhibiting AngII/TGFβ1 Fibrogenic Axis

**FIGURE 5:**
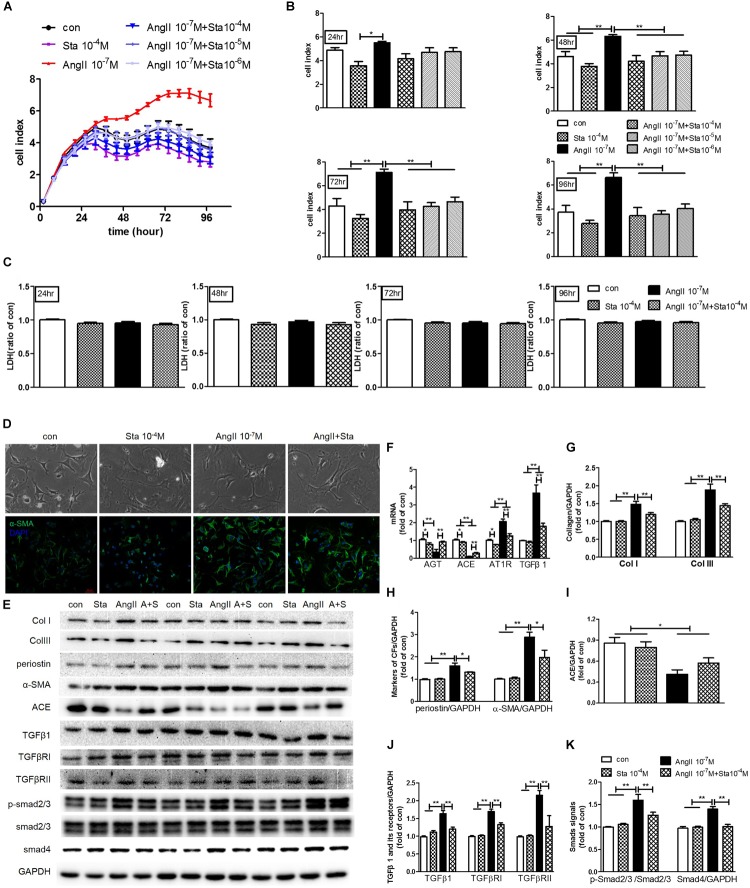
Sta suppressed AngII activated cardiac fibroblast-myofibroblast transition via inhibiting AngII/TGFβ1 fibrogenic axis. CFs were treated with or without AngII (10^−7^ M) and different dosages of Sta (10^−6^–10^−4^ M) for up to 96 h. **(A)** Representative cell proliferation curves recorded by xCELLigence TRCA system in CFs. **(B)** Statistics of cell index at 24, 48, 72, and 96 h in CFs. **(C)** Statistics of LDH content at 24, 48, 72, and 96 h in CFs. **(D)** Representative microscopic images in light field of CFs (upper row), and fluorescence (lower row) images for α-SMA and DAPI staining in CFs after drug treatments for 48 h. **(E)** Representative western blots and statistics of Col I, III, periostin, α-SMA, ACE, TGFβ1, TGFβRI, TGFβRII, phosphorylated and total Smad2/3, Smad4 and GAPDH contents in CFs after drug treatments for 48 h. **(F)** Quantitative real-time PCR of ATG, ACE, AT1R and TGFβ1 in CFs after drug treatments for 48 h. **(G–K)** Representative western blots and statistics of Col I, III, periostin, α-SMA, ACE, TGFβ1, TGFβRI, TGFβRII, phosphorylated Smad2/3, and Smad4. The data were expressed mean ± SEM. ^∗∗^*P* < 0.01; ^∗^*P* < 0.05. con, control; Sta, stachydrine; AngII, angiotensin; LDH, lactate dehydrogenase; α-SMA, α-smooth muscle actin; DAPI, 4′,6-diamidino-2-phenylindole; Col I and III, collagen I and III; GAPDH, glyceraldehyde-3-phosphate dehydrogenase; TGFβ1, transforming growth factor β1; TGFβRI, TGFβ receptor I; TGFRII, TGFβ receptor II; p-Smad2/3, phosphorylated Smad2/3; ATG, angiotensinogen; ACE, angiotensin converting enzyme; AT1R, angiotensin type 1 receptor.

To further illustrate the anti-fibrotic effect and mechanism of Sta, CF stimulated by AngII (10^−7^ M) was applied for the following experiments. Real-time cell proliferation assay demonstrated that AngII notably promoted CFs proliferation from 24 to 96 h (*P* < 0.05 or *P* < 0.01 vs. the control group, Figure 5A,B), while different dosages of Sta (10^−6^–10^−4^ M) inhibited the proliferative effect of AngII (from 48 to 96 h, *P* < 0.01 vs. the AngII group, Figure 5A,B). In addition, this effect was unrelated to the toxic effect of Sta on CFs, as evidenced by no significant changes of LDH releasing among different treatment groups at different time points (from 24 to 96 h, *P* > 0.05, Figure 5C). The representative pictures in Figure 5D further revealed that increased cell size and expression of α-SMA (marker of MFs) upon the stimulation of AngII for 48 h were inhibited by the treatment of Sta (10^−4^ M). Coincidently, the protein profiles in Figure 5E,G,H proved that AngII activated CFs by elevated expressions of Col I, III, periostin, and α-SMA (*P* < 0.01 vs. the control group), which was also significantly inactivated by Sta (*P* < 0.01 vs. the AngII group), indicating that Sta suppressed the activation and transition of CFs triggered by AngII.

To further explore the molecular mechanism underlying, the levels of AGT, ACE, AT1R and TGFβ1, together with the TGFβ1/Smads signaling pathway were explored. As shown in Figure 5F, AngII stimulation for 48 h in CFs elicited significant increment of AT1R and TGFβ1 transcripts but reduction of AGT and ACE (*P* < 0.01 vs. the control group). On the contrary, treatment of Sta not only down-regulated AT1R and TGFβ1 transcripts induced by AngII (*P* < 0.01 vs. the AngII group, Figure 5F), but suppressed the baseline levels of AGT, ACE and AT1R as well (*P* < 0.05 vs. the control group, Figure 5F). Moreover, western blotting results verified that CFs stimulated by AngII pronouncedly showed a reduction of ACE production, but more phosphorylation Smad2/3 and up-regulation of Smad4 via increased expressions of TGFβ1, TGFβRI and TGFβRII (*P* < 0.05 and *P* < 0.01 vs. the control group, Figure 5E,I–K). Meanwhile, Sta treatment suppressed the elevations of TGFβ1, TGFβRI, and TGFβRII, and thus the activation of Smads (*P* < 0.01 vs. the AngII group, Figure 5E,J,K). These data suggested that Sta forcefully inhibited AngII triggered activation and transition of CFs via suppression of *de novo* synthesis of AngII and subsequently down-regulated TGFβ1/Smads signaling pathway.

### Sta Suppressed AngII but Not TGFβ1 Activation in the AngII/TGFβ1 Fibrogenic Axis

**FIGURE 6:**
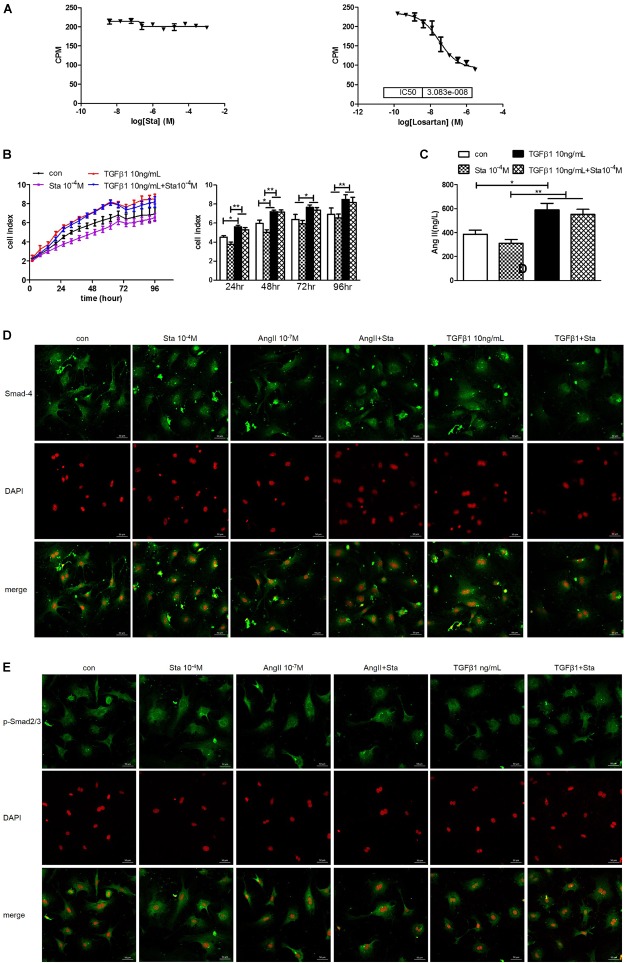
Sta suppressed AngII but not TGFβ1 activation in the AngII/TGFβ1 fibrogenic axis. **(A)** Statistics of AT1R binding assay for Sta (left) and losartan (right). **(B)** Representative cell proliferation curves recorded by xCELLigence TRCA system in CFs (left) and statistics of cell index at 24, 48, 72, and 96 h in CFs (right). **(C)** Quantification of AngII in cell culture media by ELISA. **(D)** Representative fluorescence images for Smad4 (green) and DAPI (red) staining in CFs after drug treatments for 48 h. **(E)** Representative fluorescence images for phorsphorylated-Smad2/3 (green) and DAPI (red) staining in CFs after drug treatments for 48 h. The data were expressed mean ± SEM. ^∗∗^*P* < 0.01; ^∗^*P* < 0.05. CPM, Ci/mole; con, control; Sta, stachydrine; TGFβ1, transformation growth factor 1; DAPI, 4′,6-diamidino-2-phenylindole; p-Smad2/3, phosphorylated Smad2/3; ATG, angiotensinogen; ACE, angiotensin converting enzyme; AT1R, angiotensin type 1 receptor.

The dose response curves of Sta and losartan on AT1 receptor differed greatly as shown in Figure 6A, indicating that Sta had no direct inhibitory effect on AT1R as that of the specific AT1R receptor losartan. However, considering the *in vivo* and *in vitro* results presented in Figure 4A–D, 5E,F,I which illustrated Sta significantly reduced the AGT and ACE levels upon pathological stimuli, it could be speculated that the *de novo* production of AngII by AGT/ACE could be the potential target of Sta.

To distinguish the direct target of Sta on AngII/TGFβ1 fibrogenic axis, the following experiments were further performed. TGFβ1 (10 ng/mL) incubation significantly enhanced CFs proliferation from 24 to 96 h (*P* < 0.05 or *P* < 0.01 vs. the control group, Figure 6B), but Sta showed no inhibitory effect on that (*P* > 0.05 vs. the TGFβ1 group, Figure 6B). In addition, AngII production in CFs stimulated by TGFβ1 was significantly up-regulated (*P* < 0.05, vs. the control group, Figure 6C), which could not be blunted by Sta (*P* > 0.05, vs. the TGFβ1 group, Figure 6C). More importantly, the immunofluorescence images in Figure 6D,E illustrated that the fluorescent signals of Smad4 and phosphorylated Smad2/3 at periphery of the nucleus in the control group switched to homogeneous signals inside the nucleus after AngII or TGFβ1 incubation for 48 h, indicating that both AngII and TGFβ1 stimulations significantly enhanced nuclear translocation of Smad4 and phosphorylated Smad2/3. Although Sta profoundly reversed the nuclear translocation of Smads in AngII stimulated CFs, but still failed to inhibit the activation of Smads triggered by TGFβ1 (Figure 6D,E). These data verified that Sta suppressed AngII but not TGFβ1/Smads activation in the AngII/TGFβ1 fibrogenic axis.

## Discussion

**FIGURE 7:**
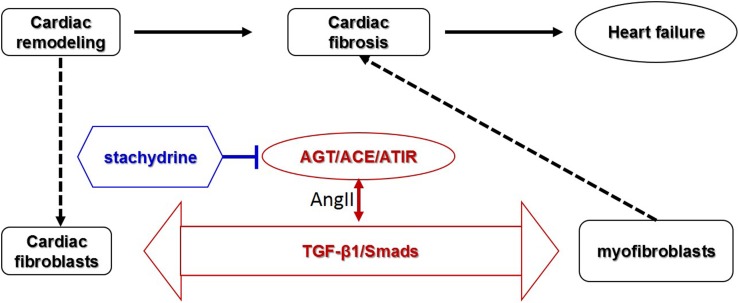
Schematic representative diagram. Stachydrine suppressed ACE/AngII/AT1R-TGFβ1 profibrotic axis, especially on the *de novo* production of AngII via down-regulating AGT/ACE and AT1R, and therefore inactivated CFs and blunted MFs transition, which ultimately prevented cardiac fibrosis.

Cardiac fibrosis is an important pathological process involved in most myocardial diseases. In this study, *in vivo* and *in vitro* experiments were applied to investigate the effect and mechanism of Sta against cardiac fibrosis. The findings in the animal experiments demonstrated that Sta treatment obviously reduced collagen deposition and fibrotic makers in pressure overload induced cardiac remodeling model, which contributed to cardiac morphological and functional improvement. The cell culture data further proved that Sta suppressed AngII activated CFs by inhibiting cell proliferation, collagen synthesis, and differentiation to MFs. Moreover, the overall mechanism underlying in this study is that Sta down-regulated *de novo* myocardial AngII production, suppressed ACE/AngII/AT1R-TGFβ1 profibrotic axis, and therefore inactivated CFs by blunting MFs *trans*-differentiation, which ultimately prevented cardiac fibrosis (Figure 7).

Sta, an active component of the Chinese motherwort *Leonurus heterophyllus* Sweet, was reported to exert multiple cardiovascular protective effects. Abundant evidences have provided extensive protective effects of Sta, including prevention of endothelial dysfunction insulted by homocysteine, high glucose, and anoxia-reoxygenation, and alleviation of myocardial hypertrophy induced by norepinephrine and pressure overload (Yin et al., 2010; Guo et al., 2012; Servillo et al., 2013; Zhang et al., 2014; Cao et al., 2017; Xie et al., 2018). Recent studies by Zhao et al. (2017) and our group have reported that Sta protected heart from isoproterenol or pressure overload induced cardiac remodeling by suppressing myocardial fibrosis (Chen et al., 2017). Moreover, this anti-fibrotic effect of Sta was also discovered in a carbon tetrachloride induced hepatic fibrosis animal model (Zhang et al., 2018). In consistence with these findings, this study demonstrated that administration of Sta potentially ameliorated TAC induced myocardial hypertrophy, notably lessened left ventricular diastolic dysfunction, and reversed both interstitial and perivascular cardiac fibrosis, which led to the improvement of left ventricular function and subsequent pulmonary congestion. However, it should be noticed that various factors, including cell injuries, necrosis, inflammation, reactive oxygen stress, hemodynamic load, and etc., contribute to the development of cardiac fibrosis (Wu et al., 2017). Therefore, the overall cardiac protective effect of Sta against pressure overload induced cardiac remodeling derives not only from the intervention of cardiac fibrosis, but from the multiple beneficial effects of Sta on the injured cardiomyocytes and endothelial cells as well.

Pathophysiological myocardial fibrosis is the result of the actions of MFs *trans*-differentiated from fibroblasts, which are α-SMA positive, apoptosis-resistant, and more importantly, persistently, and metabolically active (Weber et al., 2013; Weber and Diez, 2016). Moreover, MFs mediated collagen turnover is mainly regulated by their autocrine and paracrine factors, especially AngII and TGFβ1, which forms the fibrogenic axis consisting ACE/AngII/AT1R-TGFβ1 (Weber et al., 2013). AngII activation of ATR1 induces the expression TGFβ1, which is required for AngII to induce fibrosis (Campbell and Katwa, 1997; Schultz Jel et al., 2002). In this study, elevated expression of α-SMA, the marker of MFs, together with collagen content, after TAC and AngII stimuli was sharply blunted after Sta treatment. Both AngII and TGFβ1 significantly increased CFs proliferation, however, Sta only exerted its anti-proliferative effect against AngII but not TGFβ1. Moreover, it was illustrated that TAC also significantly enhanced myocardial production of AngII as evidenced by increased levels of AGT, ACE and AngII, while the application of Sta strongly suppressed local AngII content as well as AGT and ACE. In parallel with this, increased transcript of TGFβ1 but not the protein level of it after TAC was down-regulated by Sta. The *in vitro* experiments further proved that Sta specifically inhibited this fibrogenic axis by suppressed AngII enhanced AT1R and TGFβ1, resulting in less proliferation and blunted *trans*-differentiation of CFs triggered by AngII. Considering the *in vivo* and *in vitro* actions of Sta on the AGT/ACE/AngII/AT1R axis, we assumed that Sta exerted its inhibitory effect on the *de novo* production of AngII by down-regulating AGT/ACE and AT1R.

Numerous evidences have established the beneficial effect of anti-fibrotic strategies. ACEIs and AT1R blockers, have already showed significant efficacy in the treatment of heart failure by reducing cardiac fibrosis in human and in animal models (Varo et al., 1999; Brilla et al., 2000; Ciulla et al., 2004; Tyralla et al., 2011; Muller et al., 2013). In this study, Telmisartan exhibited pronounced anti-fibrotic effect in pressure overload mouse model, which was associated with down-regulation of AT1R and TGFβ1 mRNA transcripts, and subsequently attenuated TGFβ1 signaling pathway. This effect could be explained by the direct blocking effect of Tel on AT1R, which also lead to the elevation of AGT and ACE by a negative feedback loop (Ruilope et al., 2005; Wang et al., 2016). The application of Sta not only significantly decreased mRNA transcript of TGFβ1, but AGT, ACE and AT1R as well, implying more extensive effect on the ACE/AngII/AT1R-TGFβ1 fibrogenic axis. It should be noted that Sta had no direct inhibitory effect on the AT1R receptor. Thus, the actual pharmacological effect of Sta on the biosynthesis of AngII and TGFβ1 still needs more experimental proofs, especially the specific transcriptional targets and the signaling pathways of Sta in modulating AGT, ACE, and TGFβ1 in CFs.

TGFβ1 is clearly an important fibrotic mediator and effector in the fibrotic process via the canonical Smads and non-canonical MAPKs signaling pathways, which finally enhanced the transcriptional control of profibrotic related genes (Nattel et al., 2008; Stempien-Otero et al., 2016). Upon the activation of TGFβ1, the Smads dependent pathway starts from the phosphorylation of Smad2/3, forming complexes by binding with Smad4, and thereafter translocation to the nucleus (Biernacka et al., 2011). In this study, we identified that elevation of TGFβ1 mRNA transcript after TAC, and subsequent activation of TGFβRII/Smads, was significantly blunted by Sta and Tel treatment. Moreover, the inhibitory effect of Sta on TGFβ1 and its signaling pathway was also observed AngII stimulated CFs. Although the protein level of TGFβ1 kept unchanged in all the animal groups, it should be noted that the latent form of it in the matrix probably conceals the actual change of the activated TGFβ1 (Wipff et al., 2007), which was illustrated in *the vitro* experiment that Sta significantly reduced TGFβ1 level in CFs stimulated by AngII. Interestingly, Sta exerted no direct inhibitory effect against TGFβ1 stimulated CFs proliferation and Smads activation. These findings revealed that intervention of TGFβ1 expression might be the candidate target of Sta.

## Conclusion

In conclusion, our study demonstrated that the inhibitory effects of Stachydrine on ACE/AngII/AT1R-TGFβ1 fibrogenic axis play a pivotal role in prevention of pathological cardiac fibrosis, which further provided a strong experimental proof that Sta is a promising candidate for treating cardiac fibrosis.

## Ethics Statement

This study was carried out in accordance with the recommendations of “National Institutes of Health guide for the care and use of laboratory animals (NIH Publications No. 8023, revised 1978).” The protocol was approved by the “Animal Care and Use Committee of Shanghai University of Traditional Chinese Medicine.”

## Author Contributions

XL, XS, and ZL performed the cellular and molecular experiments. HC, PZ, and CZ performed the animal experiments. WG performed the statistical analysis. MX and RL designed and wrote the manuscript.

## Conflict of Interest Statement

The authors declare that the research was conducted in the absence of any commercial or financial relationships that could be construed as a potential conflict of interest.
